# Effect of hope therapy on psychological well-being of women after abortion: a randomized controlled trial

**DOI:** 10.1186/s12888-021-03600-9

**Published:** 2021-11-30

**Authors:** Fatemeh Raphi, Soheila Bani, Mahmoud Farvareshi, Shirin Hasanpour, Mojgan Mirghafourvand

**Affiliations:** 1 Department of Midwifery, Student Research Committee, Faculty of Nursing and Midwifery, Tabriz University of Medical Sciences, Tabriz, Iran; 2 Physical Medicine and Rehabilitation Research Center, Tabriz University of Medical Sciences, Tabriz, Iran; 3 Razi Hospital, Tabriz University of Medical Sciences, Tabriz, Iran; 4 Women’s Reproductive Health Research Center, Tabriz University of Medical Sciences, Tabriz, Iran; 5 Social Determinants of Health Research Center, Faculty of Nursing and Midwifery, Tabriz University of Medical Sciences, Tabriz, Iran; 6 Department of Family Health, Social Determinants of Health Research Center, Ardabil University of Medical Sciences, Ardabil, Iran

**Keywords:** Hope therapy, Abortion, Psychological well-being, Quality of life

## Abstract

**Background:**

Giving a healthy birth plays a vital role in a person’s personality development, but giving unhealthy birth and abortion can increase the risk of a range of mental disorders and psychological harms including reduced well-being and quality of life. Psychological interventions can reduce the problems of individuals, so the present study was conducted to evaluate the effect of counseling with hope therapy on psychological well-being (primary outcome) and quality of life (secondary outcome) of women with an experience of abortion.

**Method:**

This randomized controlled trial was conducted in Alzahra and Taleghani educational and medical centers in Tabriz, Iran, on 52 women aged 16 years and above whose pregnancies led to abortion in 2020–21. Participants were assigned to intervention and control groups using random blocking method. The intervention group received counseling with hope therapy approach in 8 sessions of 45 min twice a week. The psychological well-being and WHO Quality of Life (WHOQOL) questionnaires were completed before the intervention and immediately and 4 weeks after the intervention. Independent t-test and repeated measures ANOVA were used to compare the outcomes in two groups.

**Results:**

After the intervention based on the repeated measures ANOVA test and by adjusting the baseline score, the mean total score of psychological well-being in the counseling group was significantly higher than that in the control group (adjusted mean difference (AMD): 76.76; 95% confidence interval (95% CI): 63.81 to 89.70; *P* < 0.001). Also, the mean total score of quality of life in the counseling group was significantly higher than in the control group (AMD: 7.93; 95% CI: 6.38 to 9.46; *P* < 0.001). The mean score of all sub-domains of psychological well-being and quality of life in the counseling group was significantly higher than that in the control group (*P* < 0.05).

**Conclusion:**

Using hope therapy can improve the psychological well-being and quality of life of women after abortion. However, further clinical trials are required before making a definitive conclusion.

**Trial registration:**

Iranian Registry of Clinical Trials (IRCT): IRCT20120718010324N60. Date of registration: 17 Dec 2020. Date of first registration: 20 Dec 2020.

## Background

Fertility and pregnancy are natural stages in a couple’s life and include the concept of continuing the process of life and immortality for human beings [1]. Abortion is the most common pregnancy loss, so that 42 million women in the world experience abortions each year [2]. Abortion refers to terminating pregnancy spontaneously (miscarriage) or intentionally (induced abortion) before the fetus reaches sufficient development to survive before the twentieth week of pregnancy or weighs less than 500 g at birth [3]. Miscarriage generally occurs due to fetal chromosome aberration, maternal chronic diseases, environmental factors or accidental trauma. Induced abortions are categorized into two subcategories of elective and therapeutic abortions [4]. In Iran, abortion is limited to therapeutics indications as defined by the law which follows Islamic regulations. The law permits abortion if the fetus has severe abnormalities or if the mother’s life is in danger. However even in the case of illegal abortions, post-abortion care is available in hospitals as part of primary health care [5].

Abortion is perceived as a traumatic experience affecting a woman in contact with the health care services [6]. In addition to medical complications, more attention is being paid to psychological consequences related to abortion [7]. The findings of studies on post-abortion psychological consequences experienced by women are inconclusive. Some of the researches does not confirm an increased prevalence of psychological consequences [8, 9]. However, the results of a recent comprehensive literature review showed that experience of abortion directly contributes to mental disorders for at least some women [10]. The psychological consequences of abortion is also affected by the past history of mental illness, wanted or unwanted pregnancy, attitudes towards pregnancy and social support [11, 12]. The results of a study among post-abortion care seeking women in Tehran-Iran showed that at least one-third of the respondents have experienced psychological consequences. Thus, abortion either as a miscarriage or as an induced termination of a fetus life causes physical, psychological, and social consequences which may last for a long period of time, and affect personal, family, and social life of women [13].

Psychological well-being is the “perception of positive encounters with existing life challenges.” Psychological well-being requires an understanding of the existential challenges of life, one of which is abortion [14, 15]. Considering the important role of well-being in various aspects of psychosocial and even physical life, it is obvious that each of these dimensions and its components (feeling of independence, mastery of the environment, personality growth, positive relationships with others, having a goal life and self-control) will also play an important role in improving a person’s mental and social status [14].

There are various treatments for mental disorders including pharmacological or psychological treatments [16]. One of these psychological measures is hope therapy. Hope is a human mental need that gives people flexibility, vitality and increases people’s mental health [17]. Hope therapy is derived from Snyder’s theory and is based on cognitive behavioral therapy. This treatment is one of the most effective psychological structures emphasizing changes in cognitive levels, and thinking and focusing on solutions. It is used to reduce depression and anxiety and is successful in improving mental health and treatment of mental disorders. According to Snyder, hope is a learned skill that is taught through socialization in childhood. Snyder believes in the active nature of hope, which includes having a goal, planning and having the will to achieve the goal, and considering the barriers to achieve the goal and coping with these barriers [18]. Hope therapy is the third wave of therapies and is a combination of cognitive therapy, narrative therapy and solution-oriented and includes two stages of hope building and hope increasing [17]. Hopeful people are more focused on the problem and more active in solving it. People who think hopefully show less anxiety and more adaptation in the face of diagnosis and treatment. Studies suggest that hope therapy can help reduce psychological problems as a positive psychological intervention [19].

Midwives as women’s health care providers can play a vital role in helping women and their families in dealing with prenatal loss [20]. Considering that abortion can have a negative impact on women’s mental health and a negative impact on women’s lives, therefore, the aim of this study was to evaluate the effect of counseling with hope therapy approach on psychological well-being and quality of life of women with abortion.

## Methods

### Study design and participants

**Fig. 1:**
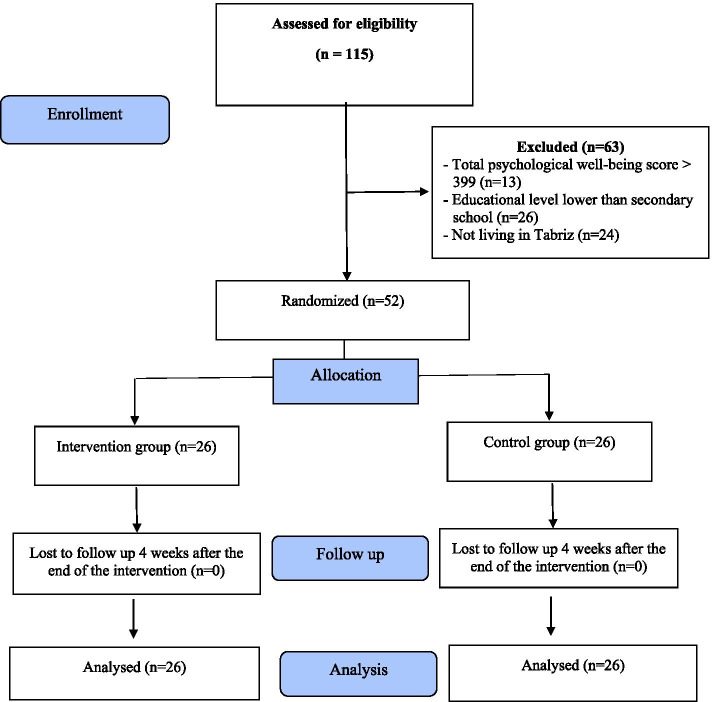
Flow chart of the study

This randomized controlled trial was conducted on 52 women aged 16 years and above who referred to Al-Zahra and Taleghani educational centers in Tabriz from December 2020 to May 2021, whose pregnancies had led to abortion. Among 115 aborted women evaluated by the researcher, 52 met the inclusion criteria. Out of 26 women assigned to the counseling group, all participated in 8 counseling sessions. There was no attrition in the study and after the interventions, 52 women were retested and the data were analyzed (Fig. 1).

Inclusion criteria included age 16 years and above, loss of pregnancy less than 20 weeks of pregnancy, minimum secondary level of education, living in Tabriz and having a contact phone number. Exclusion criteria included mental illness and use of any psychiatric medication, drug addiction, alcohol consumption, and smoking, having a history of chronic physical diseases (diabetes, heart disease, hypertension, cancer, kidney disease, lung disease, iron deficiency anemia, thyroid disease and epilepsy related diseases), experience of a severe psychological crisis during the last 3 months, such as the death of relatives and not attending more than two treatment sessions.

The sample size was calculated at 24 people based on the study conducted by Hatamloye et al. [21] and based on the psychological well-being variable and considering m_1_ = 289.1, m_2_ = 332.46 (assuming a 15% increase due to intervention), SD_2_ = SD_1_ = 40.14, two-sided α = 0.05 and Power = 95%, which considering 10% drop in the final sample size, it was considered 26 people in each group.

### Sampling

For sampling, the researcher referred to Al-Zahra and Taleghani educational and medical centers in Tabriz, and then briefly explained the goals and methods of the research to women whose pregnancies had led to abortion. If women were willing to participate in the study, they were examined in terms of inclusion and exclusion criteria and eligible individuals were selected. Then, the goals and method of conducting the research were fully explained to them and after obtaining informed and written consent, Ryff psychological well-being questionnaire (short form) was completed through interview by the researcher and participants who scored low and moderate (score less than 399) [22] on this scale and met other inclusion criteria were included in the study and completed the socio-demographic characteristics and the World Health Organization Quality of Life Questionnaire (WHOQOL) through interviews with them.

Admission of women to the hospitals for abortion was done with three chief complaints including: 1- Legal abortion with a forensic letter based on fetal anomalies (16 cases; counseling group: 9 and control group: 7); 2- Abortion due to fetal death (24 cases; counseling group: 12 and control group: 12) and 3- Inevitable abortion with complaints of bleeding and rupture of membranes (12 cases; counseling group: 5 and control group: 7). In Iran, according to the law, elective abortion is not allowed. After the women were hospitalized, all the necessary procedures were done according to the physician’s order.

### Randomization

Participants were allocated to two intervention group and control group using random blocking method stratified based on the history of abortion and the type of abortion (spontaneous or induced) with the block sizes of 4 and 6 and an allocation ratio of 1: 1. A person who was not involved in the study and was unaware of the study process determined the allocation sequence using Randomiser software. For allocation concealment, type of intervention was written on paper, placed in opaque and sealed pockets numbered sequentially. The pockets were opened in order in which the participants entered the study and the type of group of individuals was determined.

### Intervention

For the intervention group, counseling with hope therapy approach was held by the researcher (first author) and in the place intended for counseling, with a quiet and private environment in a health center. The participants were discharged from the hospital 12 to 24 h after abortion. Intervention was started for all participants in the counseling group 1 week after discharge.

The number of counseling sessions was 8 sessions. Women were given the necessary information during the sessions, and the sessions were held in an interaction between the counselor and the women. Hope therapy consultation was performed in 60–90 min sessions twice a week, in groups of 5–7 people in a place with a suitable space to observe social distance and proper air conditioning and by observing health protocols to prevent Covid-19 disease. The sessions were held using treatment plans developed by Snyder et al. (2000) [23], consisting of two main stages, including hope building and hope increasing.

#### Session 1

Pre-test, introduction and welcome, familiarity with each other, introducing the structure of the sessions, explaining the goals of the counseling program based on the theory of hope.

#### Session 2

Reviewing of the contents of the previous session, explaining how hope grows and the necessity of its existence and its effect on hopelessness, overcoming problems, depression and feelings of emptiness. Also, in these two sessions, an attempt was made to establish the necessary therapeutic relationship with each of the clients and to consider the processes and dynamics of the group.

#### Session 3

Reviewing of the contents of the previous session, each client told their life story if they wished. The goal of session was to help members find hope.

#### Session 4

Reviewing of the contents of the previous session in the group, explaining the stories based on the three main components of the theory of hope and reformatting the stories, identifying some of the hopes and past successes of the members (to identify the cause and pathways of these successes).

#### Session 5

Reviewing of the contents of the previous session, reviewing of the assignments presented in the group, a list of current events and important aspects of patients’ lives and determining the level of people’s satisfaction with each of these events.

#### Assignment

Dear friend, write down your goals and the characteristics of your goals carefully.

#### Session 6

Reviewing of the contents of the previous session, reviewing the assignments presented in the group, individuals were encouraged to select appropriate goals and to present the characteristics of appropriate goals. The goal in this session and the previous two sessions was to increase hope in the members and help the members to increase it after finding hope.

#### Assignment

Dear friend, write me the strategies you have in mind to achieve your goals (discover your own solutions).

#### Session 7

Individuals were asked to select appropriate strategies and paths to achieve the goals and were instructed to break the paths to achieve the goals into small steps and determine alternative paths. In this session, the goal was to maintain the survival of hope in the group members.

#### Session 8

Summarizing and giving feedback to the group from all the sessions, strategies for creating and maintaining the factor were proposed, including asking people to give positive self-talk and mental imagination to achieve their goals, and finally they were instructed to be a hope therapist for themselves and apply hopeful thinking on a daily basis, so that they can set their own goals and barriers, create and maintain the necessary factor to achieve that goal, and identify the necessary paths.

The control group received routine cares after abortion and the content of the consultation was provided to the control group participants after the completion of the project.

### Data collection tools

In the present study, socio-demographic and obstetrics characteristics questionnaire, psychological well-being questionnaire, and World Health Organization Quality of Life (WHOQOL) questionnaire were used to collect data before intervention (pre-test) and 8 and 12 weeks after starting the intervention (post-test) through interviews.

#### Socio-demographic and obstetrics questionnaire

This questionnaire included questions about age, educational level, sufficiency of family income level, duration of marriage, number of abortions, etc. Content and face validities were used to determine the validity of the questionnaire. The questionnaire was submitted to 10 faculty members of the university and after collecting their opinions, corrections were made on the tools based on the feedback received.

#### Psychological well-being questionnaire

This questionnaire was designed by Ryff (1989) and revised in 2002. This version has 84 questions and includes 6 components (independence, mastery of the environment, personal growth, positive communication with others, purpose in life and self-acceptance). The sum of the scores of these 6 components is calculated as the total score of psychological well-being. This test is a kind of self-assessment tool, answered on a 6-point scale from “strongly agrees” to “strongly disagree”, with a higher score indicating better psychological well-being. The correlation between the short version of the Ryff Psychological Well-being scale and the main scale ranged from 0.7 to 0.89 [24]. Khanjani et al. also examined the psychometric parameters of the Persian version this questionnaire in a study of 976 Iranian students. The results showed that the 6-factor model of this scale has a good fit. Internal consistency of this scale using Cronbach’s alpha in 6 factors of self-acceptance, master of environment, positive communication with others, having a purpose in life, personal growth and independence was calculated at 0.51, 0.76, 0.75, 0.52, 0.73, and 0.72, respectively, and for the whole scale, it was obtained at 0.71 [22].

#### WHO Quality of Life Standard Questionnaire (WHOQOL)

This questionnaire assesses individuals’ perceptions of value and cultural systems as well as their personal goals, standards, and concerns. WHOQOL is the short form containing of 26 items taken from the 100-item version of this questionnaire. The questionnaire has four subscales and a general score that measures four broad areas of physical health, psychological health, social relationships and the environment. In addition, this questionnaire can assess general health. Questionnaire items are also assessed on a 5-item scale. A higher score indicates a better quality of life [25]. Each question is scored 1 to 5, but questions 4, 3 and 26 are scored in reverse (1 = 5, 2 = 4, 3 = 3, 4 = 2, 5 = 1). Questions 1 and 2 are designed to assess clients’ perceptions of their quality of life and are not included in the scoring. Research on psychometric properties of the short form of WHOQOL questionnaire shows discriminative validity, content validity, internal reliability (Cronbach’s alpha of 0.80 for physical health, 0.76 for psychological health, 0.66 for social relationships, and 0.80 for environment) and its test-retest reliability was appropriate. The validity and reliability of the Persian version of this questionnaire in Iran have been confirmed by Nejat et al. [26].

In the present study, the reliability of the questionnaire of quality of life and psychological well-being was measured by determining internal consistency (Cronbach’s alpha coefficient), which was calculated at 0.92 and 0.88, respectively, for the psychological well-being and quality of life questionnaires.

### Data analysis

After collecting information from all participants, the data were analyzed using SPSS-24 software. The normality of the data was examined using the Kolmogorov-Smirnov test, which had a normal distribution. Independent t-test, chi-square, chi-square for trend, and Fisher’s exact test were used to examine the homogeneity of groups in terms of socio-demographic characteristics. To compare the psychological well-being and quality of life scores between the groups, before the intervention, independent t-test and after the intervention, repeated measure ANOVA test with control of baseline values and the variables of abortion number, job and husband’s job were used. *P* < 0.05 was considered significant.

## Results

The mean (SD: standard deviation) age of the participants was 30.30 (5.69) in the counseling group and 32.15 (7.41) in the control group. Table 1 shows the socio-demographic and obstetrics characteristics of participants.

**Table 1:** The socio-demographic and obstetrics charactristics of participants by study groups

Variable	Counseling group *n* = 26	Control group *n* = 26	*P*-value
	Mean (SD^*^)	Mean (SD^*^)	
**Age**	30.30 (5.7)	32.1 (7.4)	0.319^†^
**Husband’s age**	37.34 (7.6)	38.9 (7. 7)	0.472^†^
	Number (Percent)	Number (Percent)	
**Child number**			0.945^‡^
0	6 (23.1)	7 (26.9)	
1	14 (53.8)	13 (50.0)	
2	6 (23.1)	6 (23.1)	
**Gravida**			0.385^¥^
1	6 (23.1)	2 (7.7)	
2	8 (30.8)	10 (38.5)	
3	12 (46.2)	14 (53.8)	
**Parity**			0.788^‡^
0	6 (23.1)	6 (23.1)	
1	13 (50.0)	15 (57.7)	
2	7 (26.9)	5 (19.2)	
**Abortion number**			0.013^¥^
1	19 (73.1)	10 (38.5)	
2	4 (15.4)	14 (53.8)	
3	3 (11.5)	2 (7.7)	
**History of stillbirth**			1.000^¥^
no	25 (96.2)	25 (96.2)	
yes	1 (3.8)	1 (3.8)	
**Educational level**			0.091^δ^
Secondary school	5 (19.2)	7 (26/9)	
High school	3 (11.5)	2 (7/7)	
Diploma	4 (15.4)	14 (53/8)	
University	14 (53.8)	3 (11/5)	
**Job**			0.017^‡^
House keeper	17 (65.4)	24 (92/3)	
Employed	9 (34.6)	2 (7/7)	
**Husband’s education**			0.217^δ^
Primary school	2 (7.7)	3 (11.6)	
Secondary school	4 (15.4)	4 (15.4)	
High school	3 (11.5)	5 (19.2)	
Diploma	5 (19.2)	9 (34.6)	
University	12 (46.2)	5 (19.2)	
**Husband’s job**			0.004^¥^
Employed	5 (19.2)	2 (7.7)	
Laborer	3 (11.5)	15 (57.7)	
Shopkeeper	5 (19.2)	4 (15.4)	
Other	13 (50.0)	5 (19.2)	
**Sufficiency of family income for expenses**			0.437^δ^
Completely sufficient	9 (34.6)	7 (26.9)	
Relatively sufficient	5 (19.2)	4 (15.4)	
Insufficient	12 (46.2)	15 (57.7)	
**Satisfaction level of marital life**			0.128^δ^
Completely	24 (92.3)	20 (76.9)	
Partially	2 (7.7)	6 (23.1)	
Never			

*Standard deviation

^†^Independent T-test

^‡^Chi-square test

^δ^Chi-square test for trend

^¥^Fisher’s exact test

The mean (SD) of total score of psychological well-being before the intervention in the counseling group was 328.53 (42.05) which increased to 400.550 (40.59) 4 week after the intervention. In the control group, it was 326.96 (40.11) before the intervention and 315.57 (44.19) 4 weeks after the intervention. There was no statistically significant difference between the study groups before the intervention based on independent t-test (*P* = 0.891), but after the intervention, based on the repeated measures ANOVA test and with adjusting the baseline score, the mean total score of psychological well-being in the counseling group was significantly higher than the control group (adjusted mean difference (AMD): 76.76; 95% confidence interval (95% CI): 63.81 to 89.70; *P* < 0.001). Also, after the intervention, there was a statistically significant difference between the two groups in terms of all sub-domains of psychological well-being (Table 2).

**Table 2:** Comparison of the mean score of psychological well-being and its subdomains among study groups

Variable	intervention group *N* = 26	control group *N* = 26	MD (95% CI)^b^	*P*-value
	Mean (SD^a^)	Mean (SD^a^)		
**Total psychological well-being score** (Score range: 84 to 504)				
Before the intervention	328.53 (42.05)	326.96 (40.11)	1.57 (24.46 to 21.31)	0.891
Immediately after the intervention	390.38 (35.11)	319.19 (44.03)		
4 weeks after the intervention	400.50 (40.59)	315.57 (44.19)	76.76 (89.70 to 63.81)	> 0.001
**Self-acceptance** (Score range: 14 to 84)				
Before the intervention	52.92 (9.75)	53.11 (9.98)	−0.19 (5.3 to-5.69)	0.944
Immediately after the intervention	64.42 (6.35)	51.57 (10.88)		
4 weeks after the intervention	65.23 (7.56)	50.96 (10.51)	13.71 (16.07 to11.35)	> 0.001
**Environmental mastery** (Score range: 14 to 84)				
Before the intervention	53.96 (8.20)	54.42 (8.17)	−0.46 (4.10 to −5.02)	0.840
Immediately after the intervention	64.50 (8.06)	52.46 (8.35)		
4 weeks after the intervention	66.69 (7.94)	52.30 (8.90)	13.59 (16.08 to 11.11)	> 0.001
**Positive relation with others** (Score range: 14 to 84)				
Before the intervention	56.73 (8.32)	55.57 (9.52)	1.15 (6.13 to −3.83)	0.644
Immediately after the intervention	65.34 (6.29)	54.00 (9.59)		
4 weeks after the intervention	67.46 (6.96)	53.38 (9.35)	11.85 (14.45 to 9.26)	> 0.001
**Purpose in life** (Score range: 14 to 84)				
Before the intervention	56.53 (9.10)	55.50 (8.36)	1.03 (5.90 to − 3.83)	0.670
Immediately after the intervention	66.42 (7.12)	54.76 (8.86)		
4 weeks after the intervention	67.76 (8.19)	53.80 (8.96)	11.94 (14.11 to 9.76)	> 0.001
**Personal growth** (Score range: 14 to 84)				
Before the intervention	56.53 (10.56)	56.53 (8.69)	0.00 (5.39 to −5.39)	1.000
Immediately after the intervention	67.00 (7.48)	55.69 (8.52)		
4 weeks after the intervention	68.65 (7.74)	54.34 (8.50)	12.80 (15.46 to 10.15)	> 0.001
**Autonomy** (Score range: 14 to 84)				
Before the intervention	51.84 (4.81)	51.80 (4.67)	0.03 (2.68 to −2.60)	0.977
Immediately after the intervention	62.69 (4.19)	50.69 (5.18)		
4 weeks after the intervention	64.69 (5.59)	50.76 (5.45)	12.93 (15.10 to 10.76)	> 0.001

Independent t-test was used to compare the groups before the intervention and Repeated Measure ANOVA was used after the intervention with adjusting the baseline score and the variables of abortion number, job and husband’s job

^a^Standard Deviation

^b^Mean Difference (95% Confidence Interval)

The mean (SD) of the total score of quality of life before the intervention in the counseling group was 52.17 (8.89) which increased to 57.85 (7.48) 4 weeks after the intervention. In the control group, it was 48.91 (9.55) before the intervention and 47.02 (9.16) 4 weeks after the intervention. There was no statistically significant difference between the study groups before the intervention based on the independent t-test (*P* = 0.209), but after the intervention based on the repeated measures ANOVA test and adjusting the baseline score, the mean total quality of life score in the counseling group was significantly higher than the control group (AMD: 7.93; 95% CI: 6.38 to 9.46; *P* < 0.001). Also, after the intervention, there was a statistically significant difference between the two groups in terms of all sub-domains of quality of life (Table 3).

**Table 3:** Comparison of the mean score of quality of life and its sub-domains among study groups

variable	Counseling group *n* = 26	Control group *n* = 26	MD (95% CI)^b^	*P*-value
	Mean (SD^a^)	Mean (SD^a^)		
**Total quality of life score**				
Before the intervention	52.17 (8.89)	48.91 (9.55)	3.25 (8.39 to −1.88)	0.209
Immediately after the intervention	57.55 (7.40)	46.85 (9.59)		
4 weeks after the intervention	57.85 (7.48)	47.02 (9.16)	7.93 (9.46 to 6.38)	> 0.001
**Physical health**				
Before the intervention	53.62 (9.22)	51.42 (10.99)	2.19 (7.85 to −3.45)	0.439
Immediately after the intervention	58.79 (7.14)	50.21 (10.87)		
4 weeks after the intervention	59.56 (7.03)	50.32 (10.75)	7.11 (9.32 to 4.91)	> 0.001
**Psychological health**				
Before the intervention	48.84 (12.47)	45.12 (11.00)	3.71 (10.26 to −2.83)	0.260
Immediately after the intervention	56.41 (9.88)	43.07 (9.79)		
4 weeks after the intervention	56.66 (10.54)	43.46 (9.54)	10.35 (12.37 to 8.33)	> 0.001
**Social relationships**				
Before the intervention	56.41 (12.68)	53.33 (12.64)	3.07 (10.13 to −3.97)	0.385
Immediately after the intervention	60.76 (10.55)	50.76 (12.65)		
4 weeks after the intervention	60.76 (9.85)	51.02 (11.61)	7.40 (10.02 to 4.78)	> 0.001
**Environment**				
Before the intervention	49.80 (11.74)	45.76 (10.83)	4.03 (10.33 to −2.25)	0.203
Immediately after the intervention	54.23 (10.06)	43.36 (11.50)		
4 weeks after the intervention	54.42 (10.25)	43.26 (11.24)	7.37 (9.40 to 5.35)	> 0.001

Independent t-test was used to compare the groups before the intervention and Repeated Measure ANOVA was used after the intervention with adjusting the baseline score and the variables of abortion number, job and husband’s job

The total range of the quality of life and its subdomains is 0–100

^a^Standard Deviation

^b^Mean Difference (95% Confidence Interval)

## Discussion

The results of this study showed that hope therapy counseling had a positive effect on the total score of psychological well-being and its sub-domains, as well as total score of quality of life and its sub-domains.

In the present study, after the intervention, the mean total score of psychological well-being and its sub-domains in the intervention group was significantly higher than the control group. In line with the results of the present study, the results of other studies also show that counseling has a positive role in improving psychological well-being. In a quasi-experimental study conducted by Naghdi et al. [27] with the aim of determining the effect of hope therapy on psychological well-being in patients with post-traumatic stress disorder, 40 participants were included in the study and divided into intervention and control groups. The intervention group received 8 sessions of hope therapy for 2 h (two sessions per week) during 1 month, while the control group did not receive any treatment. The results revealed a significant difference between the two groups in terms of psychological well-being and hope therapy had significantly increased psychological well-being in the counseling group. In a quasi-experimental study conducted by Shokraei et al. [28] with the aim of evaluating the effectiveness of group counseling with a hope therapy approach on resilience and psychological well-being of couples, 30 couples in each group were divided into intervention and control groups. Hope therapy was established for the intervention group in weekly sessions (9 sessions of 60 min), while the control group did not receive any treatment. The results showed that after the intervention, the mean score of psychological well-being and resilience in the intervention group was significantly increased compared to the control group.

In the present study, after the intervention, the mean total score of quality of life and its sub-domains in the intervention group was significantly higher than the control group. Consistent with the results of the present study, the results of other studies also show that counseling has a positive role in improving the quality of life. In a quasi-experimental study conducted by Roientan et al. [29] with the aim of evaluating the effectiveness of combination therapy based on acceptance and commitment and hope therapy on quality of life in cancer patients. Thirty patients aged 19 to 55 years, regardless of the type of cancer, were included in the study and divided into intervention and control groups. Patients in the intervention group received 8 sessions of hope therapy counseling and the control group did not receive any treatment during this period. The results showed that the mean total score of quality of life after the counseling sessions in the intervention group was significantly higher than the control group and the effect of the intervention was 87.4%.

In a quasi-experimental study conducted by Ghezelseflo et al. [30] with the aim of evaluating the effectiveness of group counseling with a hope therapy approach in improving the quality of life of male patients with HIV, 20 men were randomly divided into control and intervention groups. The intervention group received 8 sessions of counseling with hope therapy approach and the control group did not receive any treatment. The results showed that the quality of life in the intervention group was better than the control group. The results of all mentioned study are consistent with the results of the present study.

### Strengths and limitations

Observing all the principles of clinical trial studies, including random allocation and allocation concealment were among the strengths of this study. In this study, standard questionnaires were used that psychometric properties of all of them have been assessed in Iran. Content design and counseling intervention was based on the cultural and moral values of the region. Also, no dropout was observed in the research participants and all those included in the study were analyzed. Also, mothers, due to the fear created in the area of abortion, wanted to be under the supervision of a midwife or obstetrician for re-pregnancy, which led to more cooperation in this area. The weakness of this study was its concurrence with the coronavirus pandemic and severe restrictions due to social distancing and quarantine for counseling sessions. Also, the large number of questions on the psychological well-being questionnaire was boring for individuals. Also, some of the well-being subscales have low Cronbach’s alphas suggesting that they should not be used independently. Another limitations are that sample size of this study was small and the follow-up period was very short. Similar studies are recommended for mothers who have suffered from other causes of pregnancy loss, including ectopic pregnancy, hydatidiform mole, and intrauterine fetal death (gestational age more than 20 weeks).

## Conclusion

Abortion can have negative effects on mothers’ mental health and lead to increased anxiety and stress in them and this anxiety and stress affects the physical health of mothers and can affect all aspects of their lives. The results of the present study indicate that using hope therapy counseling can improve the psychological well-being and quality of life of women after an abortion. However, further studies are needed to draw a definitive conclusion. Therefore, it is recommended to conduct clinical trials with larger sample size and longer follow-up period.

## Data Availability

The datasets used and/or analysed during the current study available from the corresponding author on reasonable request.

## Abbreviations

WHOQOL: WHO Quality of Life
AMD: Adjusted mean difference
95% CI: 95% confidence interval
IRCT: Iranian Registry of Clinical Trials
SD: Standard deviation
